# Physical and psychological burden in RRMS: depression and fatigue beyond disease duration

**DOI:** 10.1055/s-0046-1829129

**Published:** 2026-10-23

**Authors:** Neslihan Eskut, Ekin Aydin Demir, Asli Koskderelioglu, Atalay Aktuna

**Affiliations:** 1 Izmir City Hospital, Department of NeurologyIzmirTurkey; 2 Duzce Ataturk State HospitalDuzceTurkey; 3 University of Health Sciences, Izmir Faculty of Medicine, Department of NeurologyIzmirTurkey; 4 Bandirma Onyedi Eylul University, Faculty of Medicine, Department of Public HealthBalikesirTurkey

**Keywords:** Multiple Sclerosis, Disability Evaluation, Patient Reported Outcome Measures, Depression, Fatigue

## Abstract

**Background:**

Multiple sclerosis (MS) is a chronic, autoimmune, demyelinating and neurodegenerative disease. Accumulation of physical and psychological disabilities adversely affects patients' health-related quality of life. The 29-item Multiple Sclerosis Impact Scale (MSIS-29) is a self-reported measure of the physical and psychological burden.

**Objective:**

To evaluate the physical and psychological impact levels in patients with MS and to examine their relationship with disease duration.

**Methods:**

We enrolled 100 consecutive adults aged 18 to 55 years with relapsing-remitting MS (RRMS). The Expanded Disability Status Scale (EDSS), annual relapse rate (ARR), Beck Depression Inventory (BDI), Fatigue Severity Scale (FSS), MSIS-29, and Timed 25-Foot Walk Test (25-FWT) were applied.

**Results:**

A total of 57 participants (57%) were female patients. The mean age was 38.5 ± 8.0 years, and the mean disease duration, 9.8 ± 6.8 years. The mean MSIS-29 physical and psychological scores were 36.7 ± 15.7 and 45.3 ± 20.4 respectively. No significant differences were found between patients with disease duration ≤ 10 and > 10 years in terms of the physical ( *p* = 0.263) or psychological ( *p* = 0.551) subscales. Physical impact was significantly associated with the female sex ( *p* = 0.021) and the scpres on the EDSS ( *p* = 0.030), BDI ( *p* < 0.001), and FSS ( *p* < 0.001). Psychological impact showed significant associations with the female sex ( *p* = 0.005), ARR ( *p* = 0.020), and the scores on the BDI ( *p* < 0.001), and FSS ( *p* < 0.001). On multivariate regression, the scores on the EDSS, BDI, and 25-FWT were independent predictors of physical impact, while the female sex and the scores on the EDSS and BDI were independent predictors of psychological impact.

**Conclusion:**

Disease duration did not predict physical or psychological impact in RRMS. Depression severity, lower-extremity function, and the female sex were the independent determinants of disease-related impact in the multivariate analysis. The association of fatigue with disease impact, significant at the bivariate level, was attenuated by depression in the multivariate analysis. Routine assessment of these modifiable factors should be accompanied by disease-modifying treatments in MS care.

## INTRODUCTION

Multiple sclerosis (MS) is a chronic inflammatory and neurodegenerative disorder of the central nervous system that typically manifests in early adulthood and can lead to substantial, cumulative neurological disability over time. 1 In relapsing-remitting MS (RRMS), neurological deficits arising from acute attacks may persist despite effective treatments, and the ensuing physical and psychological burden exerts a negative impact on health-related quality of life. 2

The Expanded Disability Status Scale (EDSS) is the main tool which quantifies neurological impairment and tracks disease progression in MS. In clinical practice, objective clinical evaluation in MS frequently relies on the EDSS. 3 Nevertheless, it is known that the EDSS has well-recognized limitations, especially in terms of capturing the broader functional and subjective impact that MS exerts on patients' daily activities. Complementary tools that reflect the patient's own experience of their condition are therefore an essential part of comprehensive clinical evaluation. 4

The 29-item Multiple Sclerosis Impact Scale (MSIS-29) is a self-administered measure specifically designed to capture the physical and psychological dimensions of MS-related burden. 5 It comprises 20 items addressing physical function and 9 items addressing psychological wellbeing; higher scores reflect a greater degree of disease impact. Patient-reported instruments of this kind enable clinicians to access aspects of the disease burden that cannot be derived from neurological examination or imaging alone. 6

The primary aim of the current study was to assess the levels of physical and psychological impact in adults with MS using the MSIS-29, and to investigate whether disease duration independently predicts these impact scores.

## METHODS

The current is a cross-sectional study. The participants were enrolled from the Multiple Sclerosis and Other Demyelinating Diseases Outpatient Clinic of İzmir Bozyaka Training and Research Hospital, University of Health Sciences, over a 6-month period (January 11, 2023–May 1st, 2024). We enrolled patients aged between 18 and 55 years who were diagnosed with RRMS according to 2017 McDonald Criteria. The required sample size was calculated using the G*Power (free) software, version 3.1.9.7. Based on a two-tailed test with an Alpha (α) error probability of 0.05 and a statistical power of 80% to detect a moderate correlation (r = 0.30) between clinical variables and MSIS-29 scores, a minimum of 84 participants was required. To account for potential missing data and invalid questionnaire responses, and to enhance the overall statistical power, the calculated sample size was increased by approximately 20%, and 100 consecutive patients were enrolled consecutively in order of their clinical admission. The medications of the participants were listed in decreasing order of frequency, as follows: Dimethyl fumarate (25), fingolimod (22), cladribine (19), teriflunomide (13), ocrelizumab (10), interferons (6), natalizumab (2), glatiramer acetate (2), alemtuzumab (1), rituksimab (1), none (1). We excluded MS patients if they had experienced an MS relapse within the previous 3 months, severe visual or auditory impairment, had received a diagnosis of severe depression, or had any comorbid neurological or psychiatric disease. All examinations were performed in the same timeframe (09:00–12:00), with the patients required to rest for at least 15 minutes before testing began.

Sociodemographic and clinical data were recorded at the evaluation. The annual relapse rate (ARR) was derived by dividing the total number of documented relapses by the patient's disease duration in years. Neurological disability was scored using the EDSS by a single neurologist (EAD) to ensure consistency. The Beck Depression Inventory (BDI), Fatigue Severity Scale (FSS), MSIS-29, and Timed 25-Foot Walk Test (25-FWT) were then administered sequentially to each participant.

### Expanded Disability Status Scale (EDSS)

The EDSS is a clinician-rated ordinal scale, ranging from 0 (normal neurological examination) to 10 (death due to MS), used to quantify the degree of neurological disability in research and clinical contexts. 3

### Beck Depression Inventory (BDI)

The BDI is a 21-item self-report instrument that assesses the severity of depressive symptoms across cognitive, affective, and somatic domains. Respondents rate each item from 0 to 3, resulting in a total score of 0 to 63. The score thresholds are as follows: 0 to 9 –minimal or no depression; 10 to 18 – mild-to-moderate depression; 19 to 29 – moderate-to-severe depression; and 30 to 63 – severe depression. The BDI is among the most widely-recommended screening tools for depression in MS populations. 7

### Fatigue Severity Scale (FSS)

The FSS is a validated 9-item questionnaire in which each item is rated on a 7-point Likert scale (1–7). The total score is expressed as a mean across all items; values lower than 2.8 are considered within the normal range, whereas scores exceeding 6.1 are indicative of severe, chronic fatigue. 8

### 29-item Multiple Sclerosis Impact Scale (MSIS-29)

As aforementioned, the MSIS-29 is a disease-specific, patient-reported outcome tool comprising 29 items divided into a 20-item physical subscale and a 9-item psychological subscale. 5 Both subscales are scored using the standard 0-to-100 transformation: Transformed score = ([raw score - minimum possible score]/[maximum possible score - minimum possible score]) × 100. For the physical subscale, this yields a range of 0 to 100 (raw range: 20–80), and for the psychological subscale, also a range of 0 to 100 (raw range: 9–36). 9 10 Higher scores indicate a greater negative impact on daily functioning. 5 9 10

### Timed 25-foot Walk Test (25-FWT)

The 25-FWT is a performance-based measure of ambulatory function in which participants are instructed to walk a clearly-delineated 7.62-m course as rapidly and safely as possible. We ran two back-to-back trials and used the average time in seconds as the final result. 11 12

### Statistical analysis

Statistical analyses were performed using the IBM SPSS Statistics for Windows (IBM Corp.) software, version 24.0. The normality of data distribution was evaluated using the Kolmogorov-Smirnov and Shapiro-Wilk tests, along with visual inspection of histograms. All continuous variables were expressed as mean, standard deviation, median, minimum, and maximum values. The categorical variables were expressed as frequencies and percentages. Comparisons between two independent groups were performed using the Student's *t* -test for variables that met the assumption of normal distribution, and the Mann-Whitney U test, for variables that did not meet the normality assumption. Correlations were assessed with Spearman's rank correlation. Multivariate linear-regression models were developed to identify independent predictors of the MSIS-29 subscale scores. Predictor variables were selected based on a-priori clinical relevance and significant bivariate correlations, maintaining an adequate participant-to-variable ratio (> 10 participants per predictor). Multicollinearity was rigorously checked; a conservative threshold of variance inflation factor (VIF) < 2 and tolerance > 0.50 was applied. Values of *p* < 0.05 were considered statistically significant. For the sex variable, the female sex was coded as 0, and the male sex, as 1, with the female sex serving as the reference category in all regression analyses.

### Ethical approval

The study protocol was approved by the Ethics Committee of İzmir Bozyaka Training and Research Hospital, University of Health Sciences (Decision date: April 10, 2023; no: 167). Every participant provided written informed consent prior to enrolment.

## RESULTS

We enrolled 100 patients were enrolled, 57 of whom were female subjects. The mean age was 38.5 ± 8.0 (median 38.5; range: 23.0–58.0) years, and the mean disease duration was 9.8 ± 6.8 years. In total, 40 patients had a disease duration exceeding 10 years. Regarding the body mass index (BMI), 3 patients were underweight, 46 had a normal weight, 34 were overweight, and 17 were obese. Regarding the smoking status, 50 patients had never smoked, 44 were current smokers, and 6 were former smokers. The full demographic and clinical characteristics are presented in Table 1 .

**Table 1:** Demographic and clinical characteristics of the patients

Variables	Parameters	Subgroup	
Sex	n (%)	Female	57 (57.0)
		Male	43 (43.0)
Age (years)	Mean ± standard deviation		38.5 ± 8.0
	Median (minimum–maximum)		38.5 (23.0–58.0)
Marital status	n (%)	Single	28 (28.0)
		Married	72 (72.0)
Number of children	Mean ± standard deviation		1.1 ± 1.0
Years of schooling	Mean ± standard deviation		12.7 ± 4.0
Dominant hand	n (%)	Right	93 (93.0)
		Left	7 (7.0)
BMI category	n (%)	Underweight	3 (3.0)
		Normal weight	46 (46.0)
		Overweight	34 (34.0)
		Obese	17 (17.0)
Smoking status	n (%)	Never smoker	50 (50.0)
		Active smoker	44 (44.0)
		Former smoker	6 (6.0)
Disease duration (years)	Mean ± standard deviation		9.8 ± 6.8
	Median (minimum–maximum)		8.0 (1.0–27.0)
Disease duration group	n (%)	≤ 10 years	60 (60.0)
		> 10 years	40 (40.0)

The mean MSIS-29 physical score across the full cohort was 36.7 ± 15.7 (median: 33.0; range: 20.0–84.0), and the mean psychological score was 45.3 ± 20.4 (median: 42.2; range: 20.0–100.0). Contextualised against the tertile classification proposed by Jones et al. 9 in the United Kingdom (UK) MS Register ( *n* = 4,558), the mean physical score of 36.7% falls in the low-to-moderate impact range, and the mean psychological score of 45.3% falls in the moderate impact range, which is consistent with the expected burden profile of an RRMS cohort with relatively-preserved neurological function.

When the cohort was stratified by disease duration, neither the physical subscale ( *p* = 0.263) nor the psychological subscale ( *p* = 0.551) differed significantly between patients with disease duration ≤ 10 years and those with > 10 years. The physical impact scores were significantly higher among female patients ( *p* = 0.021) and those with EDSS score ≥ 3 ( *p* = 0.030). The BDI ( *p* < 0.001) and FSS ( *p* < 0.001) scores likewise showed significant univariable associations with physical impact. The psychological impact scores were significantly elevated in female patients ( *p* = 0.005) and in those with ARR ≥ 0.50 ( *p* = 0.020), as well as in individuals with higher BDI ( *p* < 0.001) and FSS ( *p* < 0.001) scores ( Tables 2 , 3 ). No significant between-group differences emerged for age, dominant hand, marital status, BMI, or smoking status.

**Table 2:** MSIS-29 physical subscale scores according to sex and EDSS scpres

Variables	Subgroup	Mean ± SD	Median (min–max)	*p*
**Sex**	Female	38.7 ± 14.4	35 (20.0–75.0)	0.021
	Male	34.0 ± 16.8	27 (20.0–84.0)	
**EDSS**	< 3	34.8 ± 13.7	32 (20.0–75.0)	0.030
	≥ 3	48.4 ± 21.5	49.5 (20.0–84.0)	
**Disease duration (years)**	≤ 10	35.4 ± 15.4	31.0 (20.0–83.0)	0.263
	> 10	38.6 ± 16.0	36.0 (20.0–84.0)	

Abbreviations: EDSS, Expanded Disability Status Scale; max, maximum; min, minimum; MSIS-29, 29-item Multiple Sclerosis Impact Scale; SD, standard deviation.

Note: Mann-Whitney U test.

**Table 3:** MSIS-29 psychological subscale scores according to sex and annual relapse rate

Variables	Subgroup	Mean ± SD	Median (min–max)	*p*
**Sex**	Female	49.3 ± 18.0	46.0 (20.0–97.8)	0.005 ^b^
	Male	40.1 ± 21.7	31.1 (20.0–100.0)	
**Annual relapse rate**	< 0.50	42.8 ± 21.0	37.0 (20.0–100.0)	0.020 ^b^
	≥ 0.50	50.7 ± 16.6	51.1 (22.2–82.2)	
**Disease duration (years)**	≤ 10	44.3 ± 18.7	41.1 (20.0–97.8)	0.551 a
	> 10	46.9 ± 22.7	43.3 (20.0–100.0)	

Abbreviations: max, maximum; min, minimum; MSIS-29, 29-item Multiple Sclerosis Impact Scale; SD, standard deviation.

Notes: ^a^ Student-t test; ^b^ Mann-Whitney U test.

The Spearman correlation analysis confirmed that the MSIS-29 physical scores were significantly and positively associated with the ARR (r = 0.220; *p* = 0.028), EDSS (r = 0.430; *p* < 0.001), 25-FWT time (r = 0.420; *p* < 0.001), BDI (r = 0.690; *p* < 0.001), and FSS (r = 0.590; *p* < 0.001). A parallel pattern was observed for the psychological subscale, which correlated positively with the ARR (r = 0.290; *p* = 0.003), EDSS (r = 0.320; *p* = 0.001), 25-FWT (r = 0.250; *p* = 0.010), BDI (r = 0.750; *p* < 0.001), and FSS (r = 0.560; *p* < 0.001). Neither disease duration, age, BMI, number of children nor level of schooling yielded a statistically significant correlation with either MSIS-29 subscale ( Table 4 ). Notably, the correlation coefficients for disease duration were r = 0.137 ( *p* = 0.176) for the physical subscale, and r = 0.047 ( *p* = 0.643) for the psychological subscale, indicating a negligible and statistically-absent relationship ( Table 4 ).

**Table 4:** Spearman correlation coefficients between MSIS-29 subscales and clinical variables

	MSIS-29 physical subscale r	*p*	MSIS-29 psychological subscale r	*p*
Disease duration (years)	0.137	0.176	0.047	0.643
Annual relapse rate	0.220	0.028	0.290	0.003
EDSS	0.430	< 0.001	0.320	0.001
25-FWT (seconds)	0.420	< 0.001	0.250	0.010
BDI	0.690	< 0.001	0.750	< 0.001
FSS	0.590	< 0.001	0.560	< 0.001

Abbreviations: 25-FWT, Timed 25-Foot Walk Test; ARR, annual relapse rate; BDI, Beck Depression Inventory; EDSS, Expanded Disability Status Scale; FSS, Fatigue Severity Scale; MSIS-29, 29-item Multiple Sclerosis Impact Scale.

In the multivariate linear regression, we identified independent predictors of both MSIS-29 subscales ( Tables 5 , 6 ). For the physical subscale, the EDSS (B = 2.589; Beta [β] = 0.222; *p* = 0.011), BDI (B = 0.958; β = 0.510; *p* < 0.001), and 25-FWT (B = 1.790; β = 0.198; *p* = 0.022) emerged as significant independent predictors. For the psychological subscale, the independent predictors were the female sex (B = -4.013; β = -0.218; *p* = 0.001), EDSS (B = 1.539; β = 0.226; *p* = 0.005), and BDI (B = 0.691; β = 0.628; *p* < 0.001).

**Table 5:** Multivariate linear regression analysis: independent predictors of the MSIS-29 physical score

	B	Standard error	Beta	t	*p*	Tolerance	VIF
Sex	-3.98	2.12	-0.127	-1.874	0.064	0.945	1.059
Age	0.07	0.16	0.034	0.430	0.668	0.687	1.456
ARR	-1.37	2.78	-0.036	-0.494	0.623	0.794	1.259
EDSS*	**2.59**	**1.00**	**0.222**	**2.589**	**0.011**	**0.587**	**1.705**
BDI*	**0.96**	**0.15**	**0.510**	**6.300**	**< 0.001**	**0.660**	**1.515**
FSS	1.26	0.68	0.153	1.848	0.068	0.634	1.577
25-FWT (seconds)*	**1.79**	**0.77**	**0.198**	**2.331**	**0.022**	**0.601**	**1.665**

Abbreviations: 25-FWT, Timed 25-Foot Walk Test; ARR, Annual Relapse Rate; BDI, Beck Depression Inventory; EDSS, Expanded Disability Status Scale; FSS, Fatigue Severity Scale; MSIS-29, 29-item Multiple Sclerosis Impact Scale; VIF, variance inflation factor.

Notes: R ^2^ = 0.61; Durbin-Watson: 2.10; sex coded as 0 = female (reference) and 1 = male.

**Table 6:** Multivariate linear regression analysis: independent predictors of the MSIS-29 psychological score

	B	Standard error	Beta	t	*p*	Tolerance	VIF
Sex (female)*	**-4.01**	**1.14**	**-0.218**	**-3.512**	**0.001**	**0.945**	**1.059**
Age	-0.09	0.08	-0.075	-1.028	0.307	0.687	1.456
ARR	0.03	1.50	0.002	0.022	0.982	0.794	1.259
EDSS*	**1.54**	**0.54**	**0.226**	**2.859**	**0.005**	**0.587**	**1.705**
BDI*	**0.69**	**0.08**	**0.628**	**8.444**	**< 0.001**	**0.660**	**1.515**
FSS	0.61	0.37	0.126	1.653	0.102	0.634	1.577
25-FWT (seconds)	-0.22	0.41	-0.041	-0.529	0.598	0.601	1.665

Abbreviations: 25-FWT, Timed 25-Foot Walk Test; ARR, annual relapse rate; BDI, Beck Depression Inventory; EDSS, Expanded Disability Status Scale; FSS, Fatigue Severity Scale; MSIS-29, 29-item Multiple Sclerosis Impact Scale; VIF, variance inflation factor.

Notes: R ^2^ = 0.68; Durbin-Watson: 1.76; sex coded as 0 = female (reference) and 1 = male.

## DISCUSSION

The central finding of the current study is the absence of any significant association involving disease duration and MSIS-29 physical or psychological subscale scores in a homogeneous cohort of RRMS patients. Instead, depression severity, fatigue, degree of neurological disability, walking performance, and the female sex emerged as the main determinants of subjective disease impact. It must be emphasized that the cross-sectional design of the present study precludes any causal interpretation of these associations; depression, fatigue, and disability may be simultaneously influenced by the same underlying disease processes, and the direction of these relationships cannot be established basaed on the present data alone. The longer time since diagnosis does not directly translate into greater subjective impact in RRMS. This may be partly due to the significantly-different natural progression of the disease under advanced disease-modifying therapies (DMTs), which have been demonstrated 1 to greatly reduce the worsening of disability in RRMS. Furthermore, the heterogeneity of DMT use in our cohort—spanning moderate- to high-efficacy agents—represents a potential confounding factor, as the level of disease control may independently influence disability accumulation, mood, and fatigue, and, therefore, patient-reported burden irrespective of disease duration.

Incorporating patient-reported outcome measures alongside clinician-rated scales has become increasingly recognized as essential for a complete understanding of the MS disease burden. 6 The MSIS-29 is a validated patient-reported outcome tool that provides information on the physical and psychological impacts experienced by patients. 5 Jones et al. 9 reported that the MSIS-29 physical scores increased modestly with disease duration, while the psychological scores remained stable. However, we did not find any relationship involving disease duration and MSIS-29 physical or psychological subscale scores. The divergence between our findings and those of Jones et al. 9 can be explained by differences in cohort composition. Jones et al. 9 enrolled patients with progressive MS subtypes, in whom a correlation between disease duration and patient-reported impact is biologically expected, given the cumulative nature of disability burden. Furthermore, our study cohort consisted of RRMS patients with relatively-preserved neurological functions evaluated in a cross-sectional design. Young et al. 10 evaluated nearly 6 thousand MS patients in a longitudinal study within 3 discrete disability impact trajectories (stable, deteriorating, and improving), and each associated substantially different MSIS-29 scores across disease duration categories. Based on the heterogeneity in these trajectories, cross-sectional studies are unlikely to demonstrate the variations in patient-reported disease impact over time.

McGuigan et al. 13 reported that the MSIS-29 physical scores reflected deterioration in EDSS scores, hence confirming the sensitivity of the physical subscale regarding objective disability change. These results align with those of our correlation analysis. Özden et al. 14 conducted a Turkish validation study of the MSIS-29 in 119 MS patients and reported that the physical subscale correlated strongly with the BDI scores and moderately with the EDSS scores. Moreover, the psychological subscale showed strong BDI correlations but only weak associations with the EDSS. These patterns closely mirror our own correlation coefficients. Håkansson et al. 15 prospectively conducted a study with 38 newly-diagnosed clinically-isolated syndrome (CIS) or RRMS patients. Fatigue scores on the MFIS were robustly associated with both MSIS-29 subscales at baseline and 1 year later, but they were unrelated to the EDSS, disease duration, lesion burden, or brain volume. The dissociation between objective neurological parameters and the perceived impact of the disease is a recurring theme reflected in our findings.

In the current study, depression exerted the strongest influence on both MSIS-29 subscales in the multivariate analysis models. The attenuation of FSS significance in the multivariate model ( *p* = 0.068 for the physical subscale; *p* = 0.102 for the psychological subscale) is consistent with the well-established overlap between BDI and FSS in MS populations. The strong correlation between BDI and FSS scores in our cohort (r = 0.590 and r = 0.560 for the physical and psychological subscales respectively) indicates shared variance that prevents independent FSS significance when both predictors enter the regression simultaneously. This does not negate the clinical importance of fatigue, but it suggests that its independent contribution may be partially mediated through depressive symptoms. Schmidt and Jöstingmeyer 16 reported that quality of life in MS was independently predicted by depression severity, fatigue, and employment status. Similarly, Aygünoğlu et al. reported that fatigue severity correlated positively with EDSS and BDI scores; sdditionally, quality of life was substantially worse among fatigued patients, but fatigue severity was not related to disease duration. 17 Yeni et al. suggested that depression has an indirect effect on quality of life, which is partly mediated by fatigue severity and sleep disturbance. 18 Our results were in line with those of the aforementioned studies.

In our cohort, female patients recorded significantly-higher MSIS-29 scores in both subscales, and the female sex emerged as an independent predictor of psychological impact. Likewise, Young et al. 10 identified substantial sex-related differences across MSIS-29 domains in a large, diverse cohort. Paradoxically, women with MS tend to follow a more favorable neurological course than men—they are less prone to primary progressive disease and accumulate disability more slowly—yet, they appear to shoulder a heavier subjective burden. Harbo et al. 19 reviewed the biological and clinical determinants of sex differences in MS, noting that the female sex is associated with a markedly-higher disease risk alongside distinct patterns of immunological reactivity and disease expression. These sex-specific biological and psychosocial factors may together underpin the greater psychological impact consistently observed among women.

The current study has several methodological strengths. First, having enrolled only RRMS patients eliminated the confounding influence of divergent pathological mechanisms across disease subtypes. Second, we applied six validated instruments which enabled us to interpret a multivariate picture of disease burden. Notably, we investigated the associations of patient-reported outcomes with disease parameters. Moreover, all patients included in the study were evaluated in the morning after resting for at least 15 minutes to eliminate the diurnal variation on fatigue and performance scores.

Several limitations to the current study should be considered. First, the cross-sectional design precludes causal inference; the associations herein reported can only be interpreted as concurrent relationships. Second, the restriction to a single-center design may limit the generalizability of findings to the larger MS population. Third, the cohort was composed exclusively of RRMS patients with a relatively-low mean disability level, which may have attenuated the range of MSIS-29 scores and reduced the statistical power to detect a duration effect. Fourth, the 10-year disease duration threshold used for the categorical analysis, while consistent with prior MS literature, 20 21 was not derived from a formal statistical method such as receiver operating characteristic (ROC) cureve analysis, and sensitivity analyses with alternative cutoffs were not performed. Fifth, the patients were receiving a heterogeneous range of DMTs spanning moderate- and high-efficacy agents; the potential differential effects of DMT category on fatigue, depression, and patient-reported outcomes were not formally analyzed as covariates, and future studies should stratify by DMT category. Sixth, patients with severe depression (BDI > 30) were excluded on clinical grounds, resulting in right truncation of the BDI distribution, which may lead to an underestimation of the BDI regression coefficient. Seventh, the ARR was calculated by dividing total documented relapses by disease duration in years; in patients with longer disease duration, relapse recall may be incomplete, potentially introducing a measurement error in this variable. Future research should employ longitudinal, multicentre designs incorporating all MS subtypes to clarify the temporal dynamics of patient-reported outcome measures and their predictors.

In conclusion, in the current study, disease duration was not associated with either physical or psychological MSIS-29 scores in patients with RRMS. Depression severity, lower extremity function, and the female sex were the independent determinants of disease-related impact in the multivariate analysis. Fatigue showed strong bivariate associations with both MSIS-29 subscales, but it did not reach independent significance in the regression models, likely reflecting its substantial overlap with depression. The results of the present study suggest that, in the current era of widely-used DMTs, the assumption that patients with a longer disease duration always experience a greater subjective disease burden is not supported in RRMS patients. These results suggest that, in the management of MS-related physical and psychological impact, systematic assessment and treatment of depression and fatigue should be implemented alongside DMTs.

### Ethical Considerations

The current study was conducted according to the ethical principles of the Declaration of Helsinki. The study protocol was approved by the Ethics Committee of İzmir Bozyaka Training and Research Hospital, University of Health Sciences (decision date: April 10, 2023; no: 167). Every participant provided written informed consent prior to enrolment.

#### Use of Artificial Intelligence

The authors declare that they did not use generative artificial intelligence (AI) in the conceptualization, data collection, analysis, or interpretation of the current manuscript. However, during the preparation of the manuscript, the authors used generative AI-assisted tools (Claude AI, Anthropic, PBC) exclusively to support language refinement and structural editing. The authors, who carefully reviewed, verified, and edited the AI-assisted content, take full responsibility for the accuracy, integrity, and originality of the final manuscript.
